# An Aptamer‐Based EXACT Anticoagulant as a Sustainable, Animal‐Free Alternative to Unfractionated Heparin

**DOI:** 10.1002/advs.202509867

**Published:** 2025-10-06

**Authors:** Haixiang Yu, George Pitoc, Manqi Zhang, Jeffrey Clancy, James Frederiksen, Amy Yan, Samuel Francis, Rebecca Sullenger, Rachel Rempel, Susannah Gammell, Jillian Caiazzi, Bruce A. Sullenger

**Affiliations:** ^1^ Department of Surgery Duke University Durham NC 27710 USA; ^2^ Department of Biochemistry School of Life Science and Technology China Pharmaceutical University Nanjing 211198 China; ^3^ Department of Medicine Duke University Durham NC 27710 USA; ^4^ Department of Emergency Medicine Duke University Hospital Durham NC 27710 USA; ^5^ School of Medicine Duke University Durham NC 27710 USA; ^6^ Departments of Pharmacology & Cancer Biology Cell Biology and Biomedical Engineering Duke University Durham NC 27710 USA

**Keywords:** anticoagulation, aptamers, cooperative effects, drug design, sustainability

## Abstract

Unfractionated heparin (UFH), designated as an essential medicine by the World Health Organization (WHO), is indispensable in cardiac surgery and various clinical applications. However, its production depends on the farming of over a billion large animals annually, posing sustainability challenges, especially amidst increasing efforts to mitigate greenhouse gas (GHG) emissions by reducing animal farming. The rising demand for UFH has outpaced its supply, and previous attempts to develop synthetic alternatives have fallen short because of their limited potency and reversibility. Here, HD1‐12dmA‐DAB is presented, a novel synthetic anticoagulant developed through chemical conjugation of a thrombin exosite‐binding aptamer (HD1) with a thrombin active‐site inhibitor (dabigatran). By optimizing dual‐site synergistic binding, HD1‐12dmA‐DAB achieves an enhancement of over three orders of magnitude in thrombin‐binding affinity and specificity compared to HD1 alone. It also demonstrates superior plasma stability and anticoagulant efficacy comparable to UFH, as validated in in vitro, in vivo, and ex vivo clotting models. This breakthrough highlights a promising step toward a sustainable, animal‐free alternative to UFH, addressing the growing clinical demand and advancing environmental sustainability objectives.

## Graphical abstract

By simply conjugating thrombin's active‐site inhibitor, dabigatran, with an exosite inhibitor, aptamer HD1, the resulting EXosite‐ACTive site (EXACT) inhibitors show two orders of magnitude higher potency and selectivity via synergistic binding, while maintaining the aptamer's antidote‐reversible trait, yielding a potential substitute for the animal‐derived heparin used in millions of heart surgeries annually.

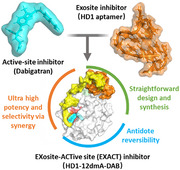

## 1 Introduction

Unfractionated heparin (UFH) is the most widely used rapid‐onset anticoagulant and has helped save millions of lives. When administered, it potentiates the endogenous anticoagulant antithrombin, which irreversibly inhibits thrombin and other coagulation factors. For the past 70 years, UFH has been an essential medicine and the gold standard anticoagulant in heart surgeries, dialysis, and other medical interventions due to its high potency and antidote‐reversibility via protamine sulfate.^[^ ^1^ ^]^

Nevertheless, the sustainability of UFH production is of increasing concern because of the growing global demand for heparin and its reliance on large‐scale pig farming. Annually, over 1.1 billion pigs are required to meet the world's need for UFH and related products such as low‐molecular‐weight heparin.^[^ ^2^ ^]^ This figure already matches the current global pig production capacity of one billion, and UFH demand is expected to increase.^[^ ^3^ ^]^ As an animal derived product, the production of UFH also generates a heavy carbon footprint: 1 gram of crude heparin is estimated to result in 23.5 to 38.1 kg of CO_2_ emissions.^[^ ^2^ ^]^ The need to expand the pig farming industry to supply life‐saving heparin conflicts with the growing recognition of the need to cut greenhouse gas (GHG) emissions by reducing large‐animal agriculture. Moreover, as the ongoing bird‐flu pandemic and the 2009 H1N1 swine flu outbreak underscore, infectious agents can rapidly destroy large numbers of domesticated animals making the supply chain of heparin insecure.

In addition to its unsustainable and vulnerable production, UFH's indirect and irreversible anticoagulation mechanism can result in variable and sometimes harmful patient responses. Furthermore, UFH's immunogenicity causes a potentially lethal condition, heparin‐induced thrombocytopenia (HIT),^[^ ^4^ ^]^ in ≈3% of patients. The intensive medical resources required for monitoring and managing UFH exacerbate the production challenges of this century‐old drug. Finding a rapid‐onset anticoagulant with comparable potency and antidote reversibility to UFH is an important unmet medical and environmental need.

Nucleic acid aptamers have recently emerged as promising, more sustainable, and secure candidates for rapid‐onset anticoagulants.^[^ ^5^ ^]^ Their direct inhibitory mechanism results in more predictable pharmacology and helps to preserve the body's capacity for normal hemostasis following reversal. Aptamers also provide high specificity, low immunogenicity, and the potential for rapid reversal using complementary oligonucleotides.^[^ ^6^, ^7^ ^]^ Unlike UFH, aptamers can be mass‐produced by chemical synthesis without GHG‐intensive and infectious agent‐susceptible large‐animal farming.^[^ ^8^ ^]^ In addition, the rapid development of RNA therapeutics has greatly improved the sustainability and scalability of oligonucleotide production.^[^ ^9^ ^]^ Yet, the inability of aptamers to inhibit protease catalytic sites limits their potency, preventing them from replacing UFH in isolation.^[^ ^5^, ^10^, ^11^, ^12^, ^13^, ^14^, ^15^, ^16^, ^17^, ^18^, ^19^, ^20^ ^]^

In an effort to increase aptamer potency and inspired by the bivalent binding mode of hirudin and other hematophagy‐derived anticoagulants, we devised a new approach to design potent anticoagulants using EXosite‐ACTive site (EXACT) inhibitors.^[^ ^21^ ^]^ This strategy involves chemically conjugating an exosite‐binding aptamer with a small‐molecule active‐site inhibitor. The synergy between the aptamer and the small‐molecule significantly enhances the binding affinity and inhibition potency of the conjugate. Despite this conceptual promise, our initial EXACT inhibitors were not as potent as heparin.^[^ ^21^ ^]^ In this study, we describe the design, creation, and evaluation of a novel EXACT inhibitor, HD1‐12dmA‐DAB, and interrogate its potential as a rapid‐onset anticoagulant in heart surgeries by using a series of in vitro, in vivo, and ex vivo clotting model systems. Our results reveal that this new EXACT inhibitor achieves 1) over three orders of magnitude higher affinity, specificity, and inhibition potency compared to the aptamer and small‐molecule inhibitor alone, 2) higher anticoagulant potency than UFH, first generation EXACT inhibitors, and other clinically used direct thrombin inhibitors such as bivalirudin, 3) sufficient stability in plasma and whole blood for rapid‐onset anticoagulation in vitro and in vivo, and 4) rapid and effective antidote‐mediated reversal. Importantly, HD1‐12dmA‐DAB is the first aptameric anticoagulant achieving UFH anticoagulation potency in an ex vivo extracorporeal membrane oxygenation (ECMO) circuit, a setting that mimics the highly coagulable state encountered during open‐heart surgeries. Thus, HD1‐12dmA‐DAB represents a potent yet controllable alternative to UFH that would not require ten billion large animals to produce over the next decade.

## 2 Results and Discussion

### 2.1 In Silico Assisted Design of the Bivalent Aptameric EXACT Inhibitor with Potent Thrombin Inhibition

**Figure 1:**
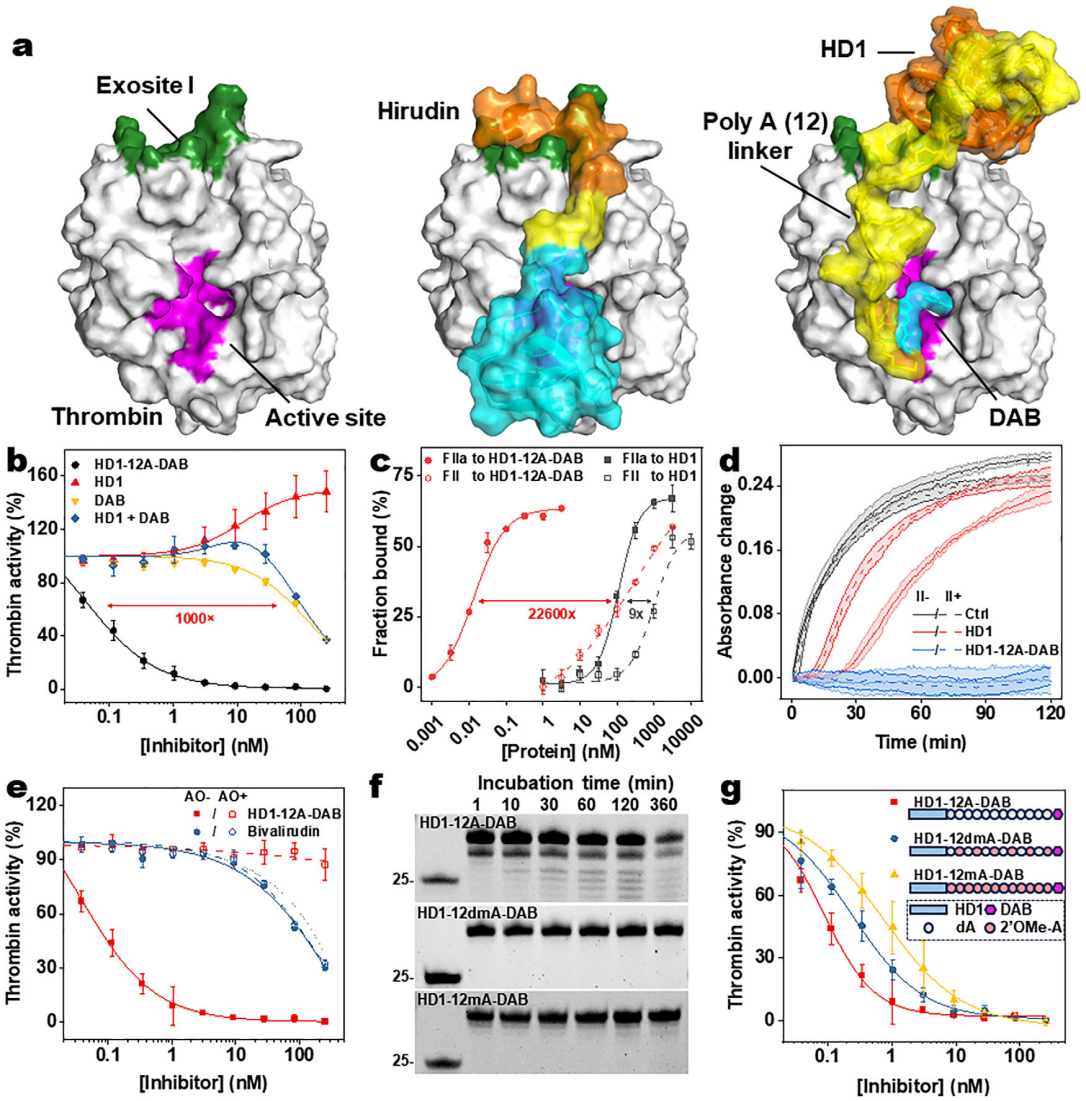
Development and characterization of aptamer hirudin HD1‐12dmA‐DAB. a) Structures of thrombin (left) and thrombin‐hirudin complex (middle) obtained from previously reported X‐ray crystallography data (PDB ID: 4HTC),^[^ ^22^ ^]^ and thrombin‐HD1‐12A‐DAB complex (right) obtained from molecular modeling. Thrombin is colored white with its exosite I colored green and its active‐site colored magenta. The active site‐binding, exosite‐binding, and linker domains of hirudin and HD1‐12A‐DAB are colored cyan, orange, and yellow, respectively. b) Thrombin inhibition of HD1‐12A‐DAB was compared with that of HD1, DAB, and their equimolar mixture in a fluorescent peptidyl substrate cleavage assay (*n* = 3), showing the high inhibitory potency of HD1‐12A‐DAB via synergistic binding. c) Binding curve of ^32^P labeled HD1 and HD1‐12A‐DAB in the presence of different concentrations of thrombin and prothrombin in a filter binding assay (*n* = 2) indicated that HD1‐12A‐DAB has a much higher thrombin binding affinity and specificity than HD1. d) Inhibition of thrombin cleavage of fibrinogen by HD1 and HD1‐12A‐DAB in the absence and presence of excessive prothrombin (*n* = 3), showing that HD1‐12A‐DAB's inhibition potency is independent of the presence of prothrombin. e) Reversal of different concentrations of HD1‐12A‐DAB by a specific antidote, AO (2 µm), in a fluorescent peptidyl substrate cleavage assay (*n* = 3). Bivalirudin was used as a control revealing its weaker inhibitory potency and lack of reversibility by AO. f,g) Plasma stability (f) and thrombin inhibition potency (g) of HD1‐12A‐DAB, HD1‐12dmA‐DAB, and HD1‐12mA‐DAB were assessed via PAGE analysis and a fluorogenic peptidyl substrate cleavage assay (*n* = 3), respectively. Data were presented as mean ± SD.

Hirudin, the major anticoagulant from leeches and one of the most potent and specific thrombin inhibitors known, comprises an active site‐binding N‐terminus and an exosite I‐binding C‐terminus (**Figure** **1**a). The synergy between the two domains enhances the thrombin binding affinity of bivalent hirudin by over 3000‐fold compared with either of its active site or exosite‐binding domains alone, achieving a 10^−11^M inhibition constant (K_i_).^[^ ^23^, ^24^ ^]^ We designed a hirudin‐like aptamer EXACT inhibitor, termed HD1‐12A‐DAB, by conjugating dabigatran, an FDA‐approved direct thrombin inhibitor, to the 3' terminus of a thrombin exosite I‐binding aptamer HD1^[^ ^25^, ^26^ ^]^ (Figure 1; Table S1, Supporting Information). A poly (12) adenosine linker was used, as suggested by molecular modeling (Figure 1a; Figure S1, Note S1, and Table S1, Supporting Information).

The thrombin‐inhibitory potency of HD1‐12A‐DAB was assessed using a fluorogenic peptidyl substrate cleavage assay (Figure 1b). In this assay, dabigatran inhibited thrombin with an IC_50_ of ≈100 nm. In contrast, HD1 binds to thrombin exosite I and does not inhibit but rather modestly enhances thrombin cleavage of the fluorogenic tripeptide substrate, possibly due to allosteric effects on the active site. The equimolar mixture of HD1 and dabigatran showed slightly lower potency than dabigatran alone. In stark contrast, HD1‐12A‐DAB potently inhibited thrombin, with an IC_50_ of 0.1 nm, 1000‐fold better than that of dabigatran, both with and without the unconjugated aptamer.

### 2.2 Synergistic Binding Mechanism Grants HD1‐12A‐DAB Exceptional Specificity

One potential limitation of HD1 is its cross‐reactivity to thrombin's zymogen prothrombin, which also presents exosite I.^[^ ^27^ ^]^ HD1 binds both thrombin (K_D_ = 110 nm) and prothrombin (K_D_ = 992 nm) with only a ninefold difference in apparent K_D_ (Figure 1c). Therefore, the effectiveness of HD1 in inhibiting trace amounts of circulating thrombin could be mitigated in the presence of excess prothrombin (1.4 µm in normal plasma). However, we found that the low selectivity of HD1 for thrombin over prothrombin can be overcome by the addition of an active site binding motif, since thrombin, but not prothrombin, presents the active site. As a result, HD1‐12A‐DAB EXACT inhibitor bound to thrombin and prothrombin with K_D_s of 13.1 pm and 296 nm, respectively, demonstrating a > 22 600‐fold selectivity for thrombin (Figure 1c). Thus, bivalent binding not only improves the affinity of the inhibitor but also enhances its specificity.

The high specificity of HD1‐12A‐DAB resulted in potent thrombin inhibition in the presence of prothrombin (Figure 1d). Thrombin (2.5 nm) rapidly cleaves fibrinogen, yielding a fibrin clot with a clotting time (t_50_) of 8 min. In the presence of aptamer HD1 (250nm), t_50_ was prolonged to 60 min, indicating strong inhibition. However, the inhibitory effect of HD1 was significantly reduced in the presence of prothrombin, with a t_50_ of 30 min. The EXACT inhibitor HD1‐12A‐DAB (5nm) markedly prolonged t_50_ to >120 min, regardless of prothrombin presence, indicating robust inhibition. The high potency and thrombin specificity of HD1‐12A‐DAB make this EXACT inhibitor an attractive candidate for rapid‐onset anticoagulation.

### 2.3 The High Potency of HD1‐12A‐DAB can be Reversed by an Antidote

We compared thrombin inhibition of HD1‐12A‐DAB to bivalirudin,^[^ ^28^ ^]^ a bivalent peptide engineered from hirudin, used in patients requiring cardiopulmonary bypass (CPB) that are allergic to UFH.^[^ ^29^ ^]^ The HD1‐12A‐DAB EXACT inhibitor was 1000‐fold more potent than bivalirudin in the thrombin cleavage assay (Figure 1e), probably because of its higher affinity. Even more importantly, unlike bivalirudin and other direct thrombin inhibitors, which lack specific reversal agents, the high potency of HD1‐12A‐DAB can be reversed by an antidote oligonucleotide (AO) (Figure 1e).

### 2.4 HD1‐12A‐DAB More Effectively Inhibits Clot‐Bound Thrombin than UFH

Despite systematic anticoagulation with UFH, thrombin bound to fibrin in clots continues to drive thromboinflammation.^[^ ^30^ ^]^ Although UFH is effective in inhibiting free thrombin, the high molecular weight of UFH‐antithrombin (ATIII) complex (≈80 KDa) makes it challenging to inhibit clot‐bound thrombin, limiting its anticoagulation effectiveness on the surface of clots.^[^ ^31^ ^]^ This spatial limitation can be observed in the fluorogenic peptidyl substrate cleavage assay (Figure S2, Supporting Information). Although the UFH‐ATIII complex (1 µm) fully inhibits free thrombin activity, it only inhibits clot‐bound thrombin by 25%. In contrast, the smaller DAB (471 Da) equally inhibited free and clot‐bound thrombin. The HD1‐12A‐DAB (1 µm) EXACT inhibitor (9.3 kDa) limited clot‐bound thrombin activity (65%) more effectively than UFH‐ATIII.

### 2.5 Linker Modification Greatly Improves Plasma Stability of HD1‐12A‐DAB

One challenge in developing DNA aptamers for therapeutic applications is their limited plasma stability. When HD1‐12A‐DAB was incubated in human plasma at 37 °C, degradation products appeared within 1 min, and most of the EXACT inhibitor was degraded within 6 h (Figure 1f). To improve plasma stability, we incorporated 2'OMe modifications into the linker backbone. Replacing all deoxy‐As with 2'OMe‐ribo‐As in the linker (termed HD1‐12mA‐DAB) resulted in enhanced plasma stability, with limited degradation observed over 6 h (Figure 1f). However, this modification also resulted in a tenfold reduction in potency (IC_50_ = 1 nm) in the fluorogenic assay (Figure 1g). Therefore, we created another derivative in which only half of the deoxy‐As in the linker were replaced with 2'OMe‐ribo‐As. This inhibitor, termed HD1‐12dmA‐DAB, showed similar plasma stability as HD1‐12mA‐DAB while preserving higher potency (IC_50_ = 0.3 nm) (Figure 1f,g).

### 2.6 EXACT Inhibitor HD1‐12dmA‐DAB is a Potent and Reversible Anticoagulant in Plasma and Whole Blood

**Figure 2:**
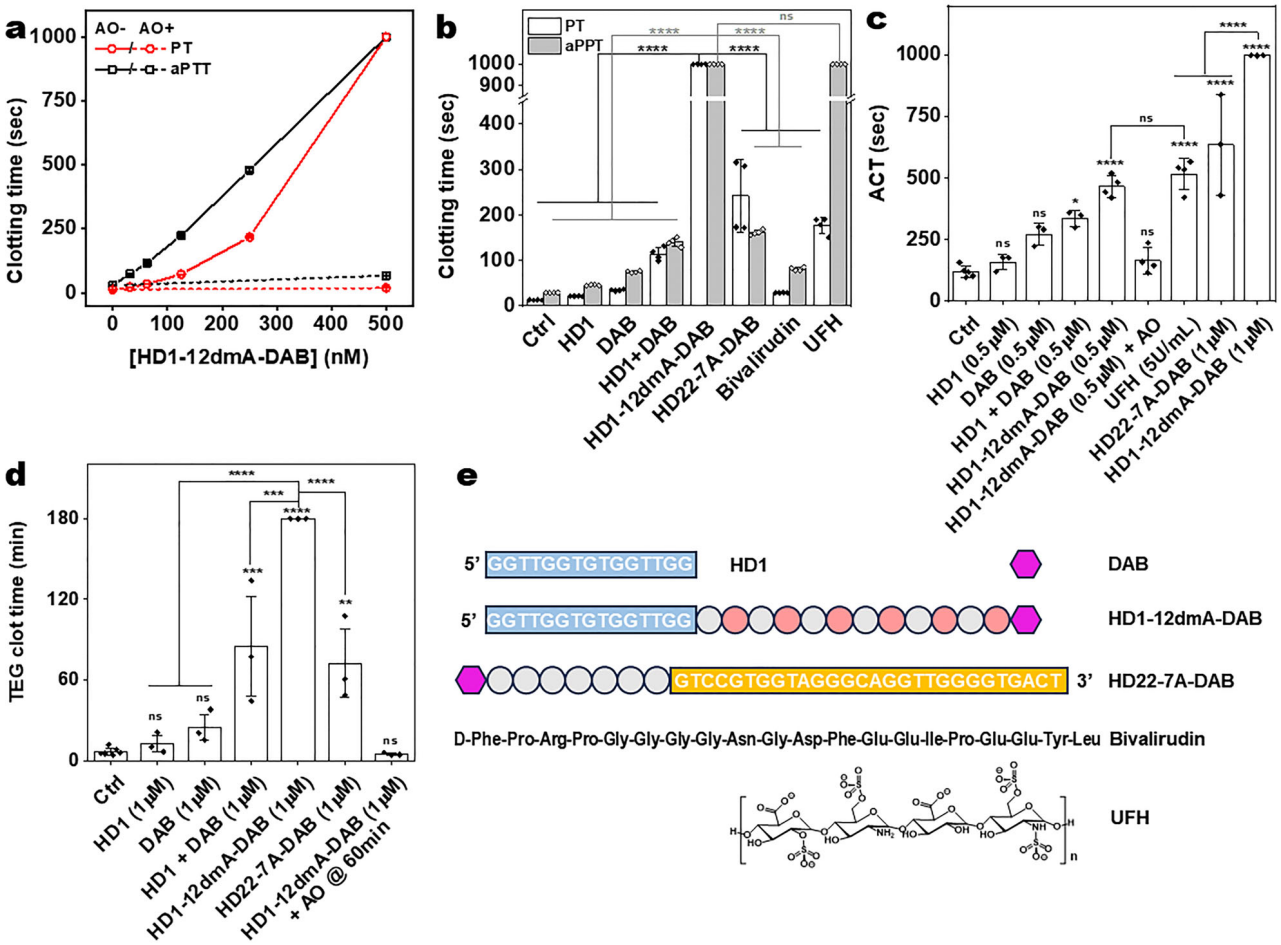
In vitro anticoagulant activity of the EXACT inhibitor HD1‐12dmA‐DAB. a) HD1‐12dmA‐DAB dose‐dependently prolonged clotting times in PT and aPTT assays in human plasma, which was reversed by AO (2 µm) addition (*n* = 4). b) HD1‐12dmA‐DAB demonstrated enhanced anticoagulant activity in PT and aPTT assays compared to UFH (5 U mL^−1^) and other anticoagulants (500 nm), (*n* = 4). c) In human whole blood, HD1‐12dmA‐DAB significantly prolonged clotting in an ACT assay (n ≥ 3), showing greater anticoagulant activity than other anticoagulants. This anticoagulation effect was reversed by AO (2 µm) addition. d) Evaluation of anticoagulation of human whole blood using TEG assay (n ≥ 3). HD1‐12dmA‐DAB was compared with other anticoagulants (1 µm). AO (2 µm) treatment rapidly restored normal clotting in HD1‐12dmA‐DAB treated blood. e) Anticoagulants used in clotting assays. Data were presented as mean ± SD. Multiple group comparisons were performed using ordinary one‐way ANOVA, followed by Tukey's post hoc test. P values were categorized in the plots as follows: >0.1234 (ns), <0.0332 (*), <0.0021 (**), <0.0002 (***), <0.0001 (****).

HD1‐12dmA‐DAB dose‐dependently prolongs plasma prothrombin time (PT) and activated partial thromboplastin time (aPTT) (**Figure** **2**a). At a concentration of 500 nm, both clotting times exceeded the maximum readout of both assays (999 seconds). This activity not only greatly exceeds those of aptamer HD1 and dabigatran alone or combined but is also significantly higher than bivalirudin and a first‐generation EXACT inhibitor HD22‐7A‐DAB that utilizes a thrombin exosite II binding aptamer (Figure 2b).^[^ ^21^ ^]^ HD1‐12dmA‐DAB also yielded a longer PT than 5 U mL^−1^ UFH, the standard heparin dose used during CPB surgery. The significantly higher anticoagulation activity of HD1‐12dmA‐DAB compared to HD22‐7A‐DAB highlights the advantage of inhibiting thrombin exosite I with the aptamer, as exosite I, which binds fibrinogen, more directly impacts fibrin generation and clotting than exosite II. Importantly, the anticoagulation activity of HD1‐12dmA‐DAB can be reversed with the antidote oligonucleotide to a near‐control level within 5 min in both clotting assays (Figure 2a).

We then evaluated the anticoagulation activity of HD1‐12dmA‐DAB in whole blood by measuring activated clotting times (ACT), a point‐of‐care assessment of anticoagulation used to monitor UFH during heart surgeries. The EXACT inhibitor HD1‐12‐dmA‐DAB (500 nm) achieved a significantly longer clotting time than the aptamer, dabigatran, or their combination (Figure 2c). Moreover, the addition of AO effectively reversed the effect of the EXACT inhibitor. When the concentration of HD1‐12dmA‐DAB was increased to 1 µm, the ACT exceeded the upper limit of the assay (999 s), which was significantly higher than the clotting time obtained with 5 U mL^−1^ UFH and exosite II‐targeted HD22‐7A‐DAB (Figure 2c).

Finally, we evaluated the anticoagulation effect of HD1‐12dmA‐DAB in a whole‐blood thromboelastogram (TEG) assay (Figure 2d; Figure S3, Supporting Information). In the absence of any anticoagulant, normal human blood had a clotting time (R‐time) of 6.8 ± 2.6 min (Figure 2d). The HD1 aptamer or dabigatran alone (1µm) modestly prolonged the clotting time to 12.6 ± 6.1 min and 24.7 ± 9.6 min, respectively, whereas their combination prolonged the clotting time to 85.2 ± 37.1 min and limited the clot stiffness (Figure S3, Supporting Information). Strikingly, HD1‐12dmA‐DAB treatment prolonged the clot time to over 180 min, the maximum time of the assay, a level that rivals 5 U mL^−1^ UFH,^[^ ^19^ ^]^ and is significantly higher than the first generation EXACT inhibitor HD22‐7A‐DAB. Such prolonged clotting in the TEG assay not only highlights HD1‐12dmA‐DAB's exceptional anticoagulation activity but also demonstrates its high stability in whole blood. To test the efficacy and kinetics of the reversal agent, we performed the same assay with HD1‐12dmA‐DAB (1 µm) but added AO (2 µm) at the 60‐min time point. Normal clotting occurred rapidly after AO addition, with a clotting time similar to that of the anticoagulant‐free blood control (4.9 ± 0.9 min, Figure 2d; Figure S3, Supporting Information). Thus, HD1‐12dmA‐DAB is a highly potent and rapidly reversible anticoagulant in whole blood that rivals or even supersedes UFH.

### 2.7 EXACT Inhibitor HD1‐12dmA‐DAB is a Potent Antithrombotic Agent in Mice

**Figure 3:**
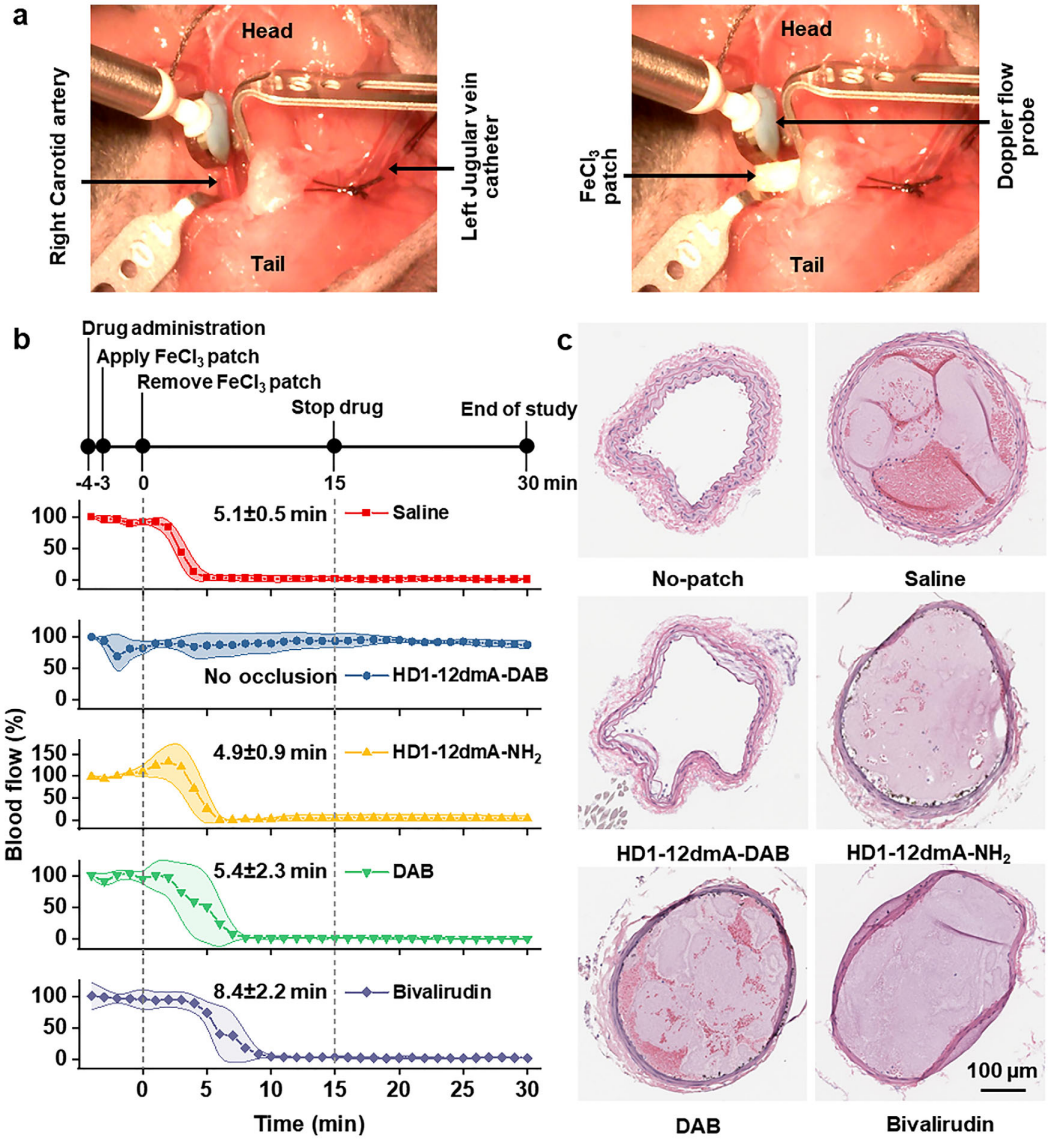
In vivo antithrombotic activity of HD1‐12dmA‐DAB. a) Experimental setup for the murine carotid artery injury thrombosis model. A Doppler flow probe was placed on the isolated right carotid artery (top panel arrow), and a ferric chloride (FeCl_3_) patch (white) was applied to induce thrombosis (bottom). b) Experimental timeline (top) and arterial blood flow under treatment with saline, HD1‐12dmA‐DAB, HD1‐12dmA‐NH_2_, DAB, or bivalirudin (*n* = 3 for each group), normalized to pre‐FeCl_3_ values. c) Representative histopathology of carotid arteries from mice treated with saline, HD1‐12dmA‐DAB, HD1‐12dmA‐NH_2_, DAB, or bivalirudin, compared to uninjured arteries (no‐patch).

The antithrombotic activity of HD1‐12dmA‐DAB was evaluated in a murine carotid artery injury‐induced thrombosis model (**Figure** **3**a).^[^ ^32^ ^]^ The right carotid artery was damaged by placing a 10% FeCl_3_ soaked filter over the artery for 3 min. In the absence of any anticoagulant, a thrombus formed and blocked blood flow in 5.1 ± 0.5 min (Figure 3b). Administration of HD1‐12dmA‐DAB prior to damage prevented vessel occlusion, as monitored by continuous blood flow over 30 min and histological analyses (Figure 3b,c). In contrast, administration of DAB or HD1‐12dmA‐NH_2_, a variant of HD1‐12dmA‐DAB in which the DAB moiety is replaced with a non‐functional amine group, did not show significant anticoagulant effect (Figure 3b,c). This result, consistent with the in vitro clotting assay, demonstrates the importance of synergistic inhibition of the exosite and the active site. Importantly, bivalirudin administration only delayed but did not prevent occlusive clot formation, with an average occlusion time of 8.4 ± 2.4 min (Figure 3b). Histological analysis of bivalirudin‐treated animals confirmed that the artery was completely occluded (Figure 3c) by 30 min. Thus, the EXACT inhibitor exhibits potent antithrombotic activity in mice.

### 2.8 HD1‐12dmA‐DAB Achieves UFH‐Rivaling and Bivalirudin‐Exceeding Anticoagulant Activity in ECMO Circuits

To closely mimic a clinically relevant setting where UFH is the standard of care and potent anticoagulation is required, we tested the ability of EXACT inhibitor HD1‐12dmA‐DAB to maintain the patency of an ex vivo ECMO circuit, which is utilized to oxygenate blood for critically ill patients or those undergoing cardiac surgery. Previously, we have observed that no single aptamer can maintain these highly procoagulant circuits clot free.^[^ ^33^ ^]^ Freshly collected blood is circulated through an oxygenator membrane with a constant flow rate (50 mL min^−1^) for 2 h while the blood pressure is monitored. The contact of blood with foreign materials and the shear force generated from the pump strongly stimulate the coagulation cascade, creating a prothrombotic challenge similar to that encountered in many open‐heart surgeries requiring CPB; thus this model is a stringent test for an anticoagulant.^[^ ^33^ ^]^

**Figure 4:**
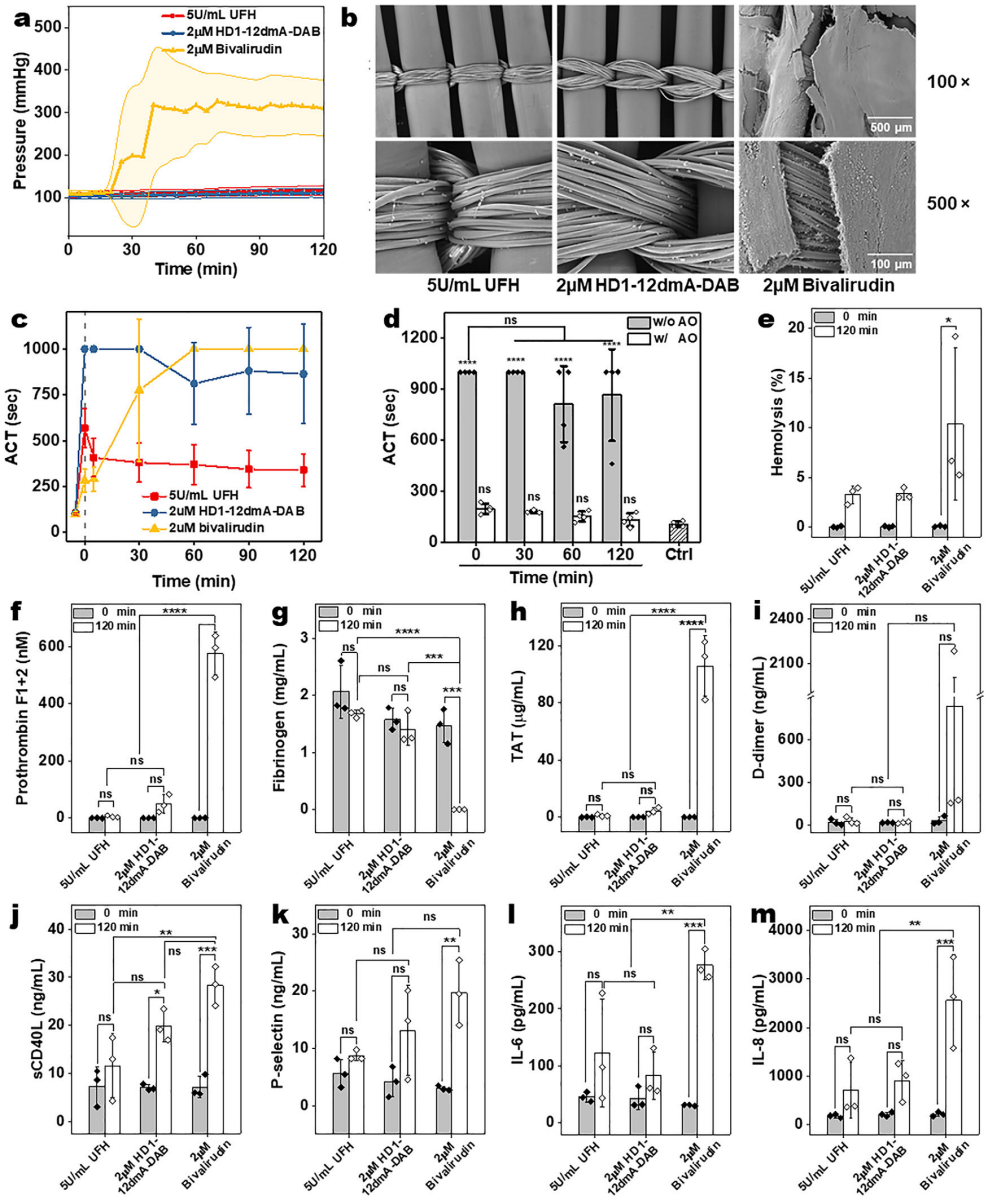
Anticoagulant activity of HD1‐12dmA‐DAB in an ex vivo ECMO circuit. a) Blood pressure during ECMO circulation with blood treated with 5 U mL^−1^ UFH, 2 µm HD1‐12dmA‐DAB, or 2 µm bivalirudin (*n *= 4 for each group). b) SEM images (100× and 500× magnification) of oxygenator membranes at the end of ECMO experiments with different anticoagulants. c) ACT measurements of blood samples collected before and during ECMO circulation with various anticoagulants (*n* = 3 for each group). d) ACT measurements of blood samples from HD1‐12dmA‐DAB‐treated circuits, further treated with AO (4 µm) for 3 min. e) Hemolysis of plasma samples before and after 2‐h ECMO circulation in each anticoagulant group (*n* = 3). f–m) Coagulation biomarkers prothrombin fragment F1 + 2 (f), fibrinogen (g), and TAT (h); fibrinolysis biomarker D‐dimer (i); platelet activation markers sCD40L (j) and p‐selectin (k); and inflammation markers IL‐6 (l) and IL‐8 (m) in plasma samples collected before and after the 2‐h ECMO circulation were measured using immunoassays (*n* = 3 per group). Data were presented as mean ± SD. Multiple group comparisons were performed using ordinary one‐way ANOVA, followed by Tukey's post hoc test. P values were categorized in the plots as follows: >0.1234 (ns), <0.0332 (*), <0.0021 (**), <0.0002 (***), <0.0001 (****).

As a positive control, blood was treated with a potent dose of UFH (5 U mL^−1^). As expected, this dose of UFH was able to maintain the ECMO circuit clot‐free at a stable pressure for at least 2 h (**Figure** **4**a). Similarly, HD1‐12dmA‐DAB (2 µm) can also maintain circuit pressure and patency for over 2 h (Figure 4a). In contrast, bivalirudin (2 µm)‐based anticoagulation is unable to maintain circuit pressure (≈100mmHg) beyond 30 min. This inability coincides with the development of large thrombi on the oxygenator membrane and in the blood reservoir in the circuit (Figure 4a,b; Figure S4, Supporting Information). Bivalirudin's ineffective anticoagulation may come from its insufficient potency, and its degradation by proteases.^[^ ^34^ ^]^ In contrast, no significant clot was observed on the oxygenator membrane or in the circuits when either HD1‐12dmA‐DAB or UFH was used as the anticoagulant (Figure 4b; Figure S4, Supporting Information).

To assess the anticoagulation of blood in the circuit more directly, ACT assays were performed on blood samples taken at different time points. With UFH (5 U mL^−1^), the ACT of the blood initially rose to 569 ± 107 s and then gradually dropped to 339 ± 88 s over 120 min. EXACT inhibitor HD1‐12dmA‐DAB (2 µm) treatment prolonged the ACT to over 999 s at the beginning of the circulation and maintained potent anticoagulation of the blood until the end of the study (ACT of 864 ± 270 s at 2 h) (Figure 4c,d). Conversely, blood treated with bivalirudin started with a much lower ACT (283 ± 61 s), but the ACT rapidly increased within 30 min, exceeding 999 s by 60 min. This increase likely results because the coagulation potential of the blood is completely depleted as large thrombi have been formed over time, which consumes many coagulation factors including prothrombin, an issue that can be problematic in the clinic.^[^ ^35^ ^]^ To confirm that prothrombin was not depleted in blood containing HD1‐12dmA‐DAB, we treated the circulated blood at different time points with AO (4 µm). Within 3 min, AO addition returned ACT levels to normal (Figure 4d). These results indicate that HD1‐12dmA‐DAB prevents the consumption of coagulation factors, and that the addition of AO can rapidly restore normal hemostasis to blood following circulation. In addition, no significant degradation of HD1‐12dmA‐DAB was observed in the samples collected from the circuit over time (Figure S5, Supporting Information). HD1‐12dmA‐DAB treatment also reduced hemolysis, a common side effect of ECMO as effectively as UFH but better than bivalirudin (Figure 4e).^[^ ^36^ ^]^

### 2.9 HD1‐12dmA‐DAB Preserves Coagulation Factors as Effectively as UFH in Blood Subject to ECMO

Finally, we measured various biomarkers for thrombin activation (prothrombin F1 + 2), thrombin activity (fibrinogen), thrombin deactivation (thrombin‐antithrombin complex, TAT), fibrinolysis (D‐dimer), platelet activation (sCD40L and p‐selectin), and inflammation (IL‐6 and IL‐8) before and after circulation in the presence of different anticoagulants using immunoassays. We found that HD1‐12dmA‐DAB (2 µm) was able to maintain the levels of thromboinflammatory markers as effectively as UFH (5 U mL^−1^) (Figure 4f–m). In contrast, most of these biomarkers are significantly altered when bivalirudin is used as an anticoagulant. Here, the significantly elevated prothrombin F1 + 2 and TAT levels (Figure 4f,h) and reduced fibrinogen levels (Figure 4g) indicate a depletion of coagulant capacity. These results correlate with the clots observed on the ECMO membranes in the bivalirudin‐anticoagulated samples (Figure 4b). They also confirmed that the unusually high ACT of bivalirudin‐treated blood samples at later time points was due to the exhaustion of clotting capacity (Figure 4c). Bivalirudin anticoagulated blood also shows higher levels of platelet activation and inflammation markers (Figure 4j–m) than blood anticoagulated with UFH or the EXACT inhibitor. In summary, the ECMO circuit data indicates that HD1‐12dmA‐DAB has a high anticoagulation potency that is at least comparable to UFH and significantly higher than that of bivalirudin in a clinically relevant, highly prothrombotic setting. Moreover, the EXACT inhibitor can match UFH's ability to preserve coagulation/anticoagulation capacity and significantly limit inflammation during ECMO. Equally important, the anticoagulant activity of the EXACT inhibitor can be rapidly and effectively reversed by an antidote oligonucleotide.

## 3 Conclusion

The unsustainable and insecure production of UFH has become a serious supply chain and environmental challenge, especially with the rising incidence of cardiovascular disease and increasing demand for potent onset anticoagulants. Unfortunately, the development of UFH replacements has been challenging, and bivalirudin, the only FDA‐approved UFH alternative used as a last resort in open‐heart surgeries, suffers from limited potency and the lack of a reversal agent.

In this study, we describe an optimized nucleic acid‐based bivalent thrombin EXACT inhibitor, HD1‐12dmA‐DAB. This second generation EXACT inhibitor retains synergy‐mediated high binding affinity, inhibitory potency, and antidote‐reversibility associated with first generation EXACT inhibitors but also has greatly improved properties. This new type of protease inhibitor reveals that: 1) The small‐molecule inhibitor domain, in addition to the aptamer domain, significantly contributes to the selectivity of the EXACT inhibitor, which allows the HD1‐based EXACT inhibitor to selectively target thrombin in the presence of high prothrombin concentrations. 2) The binding location of the aptamer on the protease, in addition to its affinity, impacts the potency of the EXACT inhibitor. 3) The low molecular weight of the EXACT inhibitor allows it to penetrate biomatrices, such as plasma clots more effectively than UFH. 4) The plasma stability of the EXACT inhibitor can be significantly improved by chemical modification of the linker domain. These properties enable the optimized EXACT inhibitor HD1‐12dmA‐DAB to achieve anticoagulant activity equal to or exceeding that of UFH and significantly surpassing that of bivalirudin and the first‐generation EXACT inhibitor HD22‐7A‐DAB in vitro, in vivo, and ex vivo. These results not only highlight the potential of HD1‐12dmA‐DAB as a potent and safe UFH alternative in hypercoagulable settings, such as ECMO and cardiopulmonary bypass surgery, but also provide guidance for the future construction of EXACT inhibitors targeting other proteases or enzymes.

Nucleic acid therapeutics have important manufacturing advantages over UFH. Aptamer‐based EXACT inhibitors can be produced on a large scale by chemical synthesis, which does not require large animals. Therefore, they promise to deliver an essential anticoagulant medicine that can replace heparin for many clinical procedures without requiring the farming of tens of billions of GHG‐producing and pathogenically vulnerable large animals in the coming decades. Several mRNA vaccines developed during the COVID‐19 pandemic have demonstrated such record‐breaking manufacturing speed and capacity.^[^ ^37^ ^]^ The main development and translational challenge for such a more sustainable medicine is that UFH is inexpensive and entrenched. Thus, it shares many qualities with the replacement of fossil fuels.

## 4 Experimental Section

### Materials

Unmodified DNA oligos were purchased from Integrated DNA Technologies. Oligonucleotides with 2'‐O‐methyl modifications were synthesized in‐house using a Mermade oligo synthesizer, followed by high‐performance liquid chromatography (HPLC) purification. UFH (1000 U mL^−1^) was obtained from Duke Hospital Pharmacy. Thrombin, fibrinogen, and the thrombin fluorogenic substrate (SN‐17a) were obtained from Prolytix. Dabigatran was acquired from AK Scientific Inc. Bivalirudin trifluoroacetate salt (SML1051), 1‐Ethyl‐3‐[3‐dimethylaminopropyl] carbodiimide hydrochloride (EDC), and N‐hydroxysuccinimide (NHS) were purchased from Sigma. PT‐excel and aPTT reagents were obtained from Diagnostica Stago. ACT cartridges (JACT+) were purchased from Werfen. TEG cups, pins, and kaolin were acquired from Haemonetics. The small animal ECMO circuit apparatus and ECMO membrane (10 cm × 10 cm) were purchased from Martin Humbs. The LegendPlex human thrombosis panel (10‐plex) (cat. 740891) and human Fibrinolysis Panel (5‐plex) (cat. 740760) multi‐analyte flow assay kits were obtained from BioLegend. Enzygnost F 1 + 2 (monoclonal) and Enzygnost TAT micro assay kits were obtained from Siemens Healthineers. Pooled normal human plasma was purchased from George King Biomedical. Human whole blood was collected from healthy male and female donors under Duke IRB protocol (Pro00007265). Murine in vivo studies were conducted using 20‐week‐old female C57BL/6 mice obtained from Jackson Laboratory.

### Ethics

This research complies with ethical regulations under Duke University Health System (DUHS) Institutional Review Board for Clinical Investigations with Federal wide Assurance No: FWA 0000902, and Duke Institutional Animal Care and Use Committee (IACUC) with protocol registry number A109‐24‐05.

### Synthesis of EXACT Inhibitor

EXACT inhibitors were synthesized as described previously.^[^ ^21^ ^]^ Briefly, EDC (1 µmol), NHS (4 µmol), and DAB (1.2 µmol) were mixed in 250 µL DMSO at room temperature for 30 min. The oligonucleotide with a 3' amine end label (5 µmol), dissolved in 60 µL triethylamine (TEA)/HCl buffer (420 mm TEA, pH 10), was then added to the mixture and incubated overnight at room temperature. The EXACT inhibitor was purified by ethanol precipitation and HPLC. The purity of the final product was confirmed by denaturing PAGE.

### Fluorogenic Peptidyl Substrate Cleavage Assay

The fluorogenic substrate assay was conducted as previously described.^[^ ^21^ ^]^ Briefly, thrombin (final concentration 0.5 nm) and the test inhibitor were mixed in 15 µL of reaction buffer (20 mm HEPES, 150 mm NaCl, 2 mm CaCl_2_, 0.02% Tween 20, pH 7.5) and loaded onto a 384‐well opaque microplate. After incubation for 5 min at 28 °C, 10 µL of fluorogenic substrate (final concentration 50 µm) was added and rapidly mixed by pipetting. The rate of fluorescence increase (λex = 352 nm, λem = 470 nm) over the first 15 min was measured using a SpectraMax i3 microplate reader (Molecular Devices). A 100% thrombin activity control was established in each experiment by measuring the fluorescence increase rate without any inhibitor. Each experiment was performed in triplicate. IC_50_ and maximum inhibition values were determined by fitting the inhibition curves using the Hill equation.

### Molecular Dynamics Simulation

The initial structures of HD1 with linker domains of varying lengths complexed with the thrombin heavy chain were constructed based on the published X‐ray crystal structure of the HD1‐thrombin complex (PDBID 4dii).^[^ ^26^ ^]^ The missing residues of the thrombin heavy chain in the crystal structure were refined using AlphaFold 3.^[^ ^38^ ^]^ The linker domains were added by extending the 3' terminus of HD1 with a B‐DNA structure using BIOVIA Discovery Studio Visualizer. The coarse‐grained structures of the complexes were then generated using the Martinize 2 tool^[^ ^39^ ^]^ with elastic network constraints on the HD1 domain and the thrombin heavy chain. A two‐step steered molecular dynamics simulation was then performed for each structure using GROMACS with the Martini3 force field.^[^ ^40^, ^41^ ^]^

In the first step, the HD1 motif was constrained to its binding site, while the 3' end of the linker was steered toward 1 nm from the active center of thrombin (centered on residues N102, I174, and G219) at a constant rate over 2 ns using umbrella pull (1000 kJ mol^−1^ nm^−2^). The linker terminus was constrained toward the thrombin active center for an additional 8 ns to equilibrate the linker domain. In the second step, while the constraint toward the active center of thrombin was maintained, the constraint on the HD1 domain at its thrombin‐binding site was removed for a 10 ns simulation. The distance between the linker terminus and the active center of thrombin, as well as the RMSD of the HD1 domain from its original structure, were recorded every 100 ps.

The all‐atom structure of the complex at the end of the simulation was back‐mapped using the backward tool.^[^ ^42^ ^]^ Dabigatran was added to the structure by superimposing the published dabigatran‐thrombin complex structure (PDBID 1kts) onto the simulated thrombin heavy chain.^[^ ^43^ ^]^

### Nitrocellulose Filter Binding Assay

The apparent dissociation constants (K_D_) of HD1 and HD1‐12A‐DAB were determined as previously reported.^[^ ^44^ ^]^ Briefly, ^32^P 5′‐end radiolabeled HD1 or HD1‐12A‐DAB (final concentration 500 CPM µL^−1^) was incubated with different concentrations of protein in the selection buffer for 5 min at room temperature. The mixtures were filtered through a nitrocellulose blotting membrane over a nylon hybridization transfer membrane to trap the protein‐bound and unbound RNA, respectively. The fraction of protein‐bound RNA was quantified using a Storm 840 phosphoimager (GE Healthcare) for K_D_ calculation. All samples were measured in duplicate.

### Fibrinogen Cleavage Assay

The fibrinogen turbidity assay was performed as previously described.^[^ ^21^ ^]^ Briefly, thrombin (final concentration 2.5 nm), prothrombin (final concentration 1250 nm), and the test inhibitor (250 nm HD1 or 50 nm HD1‐12A‐DAB) were mixed in 30 µL reaction buffer. The mixture was loaded onto a 384‐well clear‐bottom plate and incubated at 37 °C for 5 min. Then, 20 µL of fibrinogen (final concentration 0.8 mg mL^−1^) was added to the well and rapidly mixed by pipetting. The absorbance at 550 nm was monitored over 2 h using a SpectraMax i3 microplate reader. Each experiment was performed in triplicate.

### Inhibition of Clot‐Bound Thrombin

Plasma clots were prepared in a 96‐well plate by adding 20 µL of citrated pooled human plasma (George King Bio‐Medical) to 20 µL of 25 mm CaCl_2_ solution. The recalcified plasma was incubated at 37 °C for 60 min, allowing a solid clot to form at the bottom of each well. The clot was then washed every 15 min with 250 µL of reaction buffer over the course of 4 h to completely remove free thrombin. The final wash buffer was collected and analyzed using the fluorogenic substrate assay to confirm the absence of thrombin activity.

After removing all wash buffer, 50 µL of reaction buffer containing 2 µm DAB, HD1‐12dmA‐DAB, or a mixture of 2 µm ATIII and 10 U mL^−1^ UFH was added to the clot and incubated for 5 min at 28 °C. Next, 50 µL of reaction buffer containing 100 µm fluorogenic substrate was added to each well to monitor the rate of fluorescence increase. A 100% thrombin activity control was established in each experiment by measuring the fluorescence increase rate without any inhibitor. Each experiment was performed in triplicate.

### Plasma Stability Assay

The plasma stability of EXACT inhibitors was characterized as previously reported.^[^ ^44^ ^]^ The EXACT inhibitor (final concentration 500 nm) was mixed with 200 µL of plasma anticoagulated with 10 U mL^−1^ UFH and incubated at 37 °C for 6 h. Plasma samples (10 µL) were collected at various time points (1, 10, 30, 60, 120, and 360 min) into 300 µL of a water: chloroform mixture (1:1:1, v: v: v) and vortexed to deactivate nucleases. The aqueous phase containing oligonucleotides was collected and lyophilized after centrifugation at 500 rcf for 20 min at 4 °C. The sample was reconstituted in water, analyzed on a 15% polyacrylamide gel containing 7 m urea and stained with 1× SYBR gold.

### Plasma Clotting Assays

PT and aPTT assays were performed using pooled normal human plasma, as previously described,^[^ ^21^ ^]^ using an ST4 coagulometer (Diagnostica Stago). For the PT assay, 50 µL of plasma was mixed with 5 µL of the inhibitor dissolved in the reaction buffer. After 5 min, 100 µL of TriniCLOT PT Excel S reagent was added to initiate the assay. For the aPTT assay, 50 µL of plasma was mixed with 5 µL of the inhibitor dissolved in the reaction buffer. After 5 min, 50 µL of TriniCLOT aPTT S reagent was added, followed by another 5 min of incubation. Then, 50 µL of 0.2 m CaCl_2_ was added to initiate clotting. To test the antidote effect, 5 µL of AO (final concentration 2 µm) in reaction buffer was added 5 min after the EXACT inhibitor, followed by an additional 5‐min incubation. All assays were performed in quadruplicate.

### Whole Blood Clotting Assays

ACT was performed as previously described,^[^ ^21^ ^]^ using ACT+ cuvettes and a Hemochron Jr Signature coagulation analyzer. Briefly, 72 µL of freshly collected citrated whole blood was mixed with 6 µL of the inhibitors in reaction buffer at room temperature for 3 min. Then, 2.1 µL of CaCl_2_ (245 mm) was added to the mixture, and the blood sample was immediately tested for ACT. To test the antidote effect, the EXACT inhibitor and AO (final concentration 2 µm) were reconstituted together in 6 µL of reaction buffer and added to the blood sample simultaneously. All assays were performed in triplicate or more.

TEG was performed as previously described using a Thromboelastograph Analyzer (Haemonetics).^[^ ^19^ ^]^ Briefly, 320 µL of freshly collected citrated whole blood was mixed with the test anticoagulant reconstituted in 10 µL of reaction buffer and 10 µL of kaolin (Haemonetics). Then, 20 µL of 0.2 m CaCl_2_ was added, and the mixture was immediately loaded into a TEG test cup to initiate the assay. Clot formation was monitored for 180 min. To test the antidote effect, 2 µL of AO (final concentration 2 µm) was added to the blood sample 60 min after the assay began. All assays were performed in triplicate or more.

### Murine Carotid Artery Injury Thrombosis Model

The in vivo experiments were modified from a previously described method^[^ ^45^ ^]^ and approved by the Duke University Institutional Animal Care and Use Committee. Mice were anesthetized with isoflurane and tribromoethanol (Avertin) and then subjected to mechanical ventilation while positioned supine on a temperature‐monitoring board. The left external jugular vein was isolated, catheterized, and connected to a syringe pump (Harvard Apparatus, USA) for drug administration. The right common carotid artery was also isolated and fitted with a Doppler flow probe (Transonic Systems, USA). Baseline carotid flow was recorded prior to drug administration.

Mice were treated with HD1‐12dmA‐DAB, DAB, HD1‐12dmA‐NH_2_, or bivalirudin dissolved in saline (0.9% NaCl) via a bolus injection (80 nmol kg^−1^) followed by a continuous infusion (16 nmol kg^−1^ min^−1^) or with negative control (saline). One minute after the bolus injection, a 1 mm × 2 mm Whatman paper patch presoaked in 10% ferric chloride was placed on the carotid artery for 3 min. Subsequently, the patch was removed, and the injured site was rinsed with 100 µL saline. Drug infusion was stopped 15 min after patch removal, and carotid blood flow was recorded for another 15 min. At the end of the study, the tested artery was harvested for further histological analysis by Histowiz, and the animals were euthanized.

### Ex Vivo ECMO Circuit

The circuit experiments were conducted as previously described,^[^ ^19^ ^]^ using a small animal membrane oxygenator apparatus with a mechanical roller pump (MasterFlex) and a miniature 100 cm^2^ membrane oxygenator. Freshly collected blood (30 mL) was mixed with different anticoagulants and immediately introduced into the circuit for continuous circulation over 120 min, under oxygenation with 95% O_2_ and 5% CO_2_ gas. Blood pressure and flow were monitored throughout the experiment. Blood samples were collected before starting the circuit and at 5, 30, 60, 90, and 120 min after circulation commenced to measure ACT. Plasma samples were prepared from these blood samples using 3.2% sodium citrate as an anticoagulant, followed by centrifugation at 1800 rcf for 20 min.

### Hemolysis

Hemolysis during the ECMO circuit study was assessed using plasma samples collected at different time points, as previously reported.^[^ ^46^ ^]^ Briefly, plasma samples were diluted 2× with 1× PBS, and the absorbance of hemoglobin was measured at 571 nm using a NanoDrop. Hemolysis percentages were calibrated using 0% and 10% hemolysis reference samples prepared from citrated blood collected from the same donor on the same day of study. The 0% hemolysis sample was prepared by centrifuging citrated blood at 1800 rcf for 20 min and diluting the plasma 2× with 1× PBS. The 10% hemolysis sample was prepared by mixing citrated blood with an equal volume of deionized water for 30 min, followed by centrifugation at 1800 rcf for 20 min, and diluting the plasma 10× with the 0% hemolysis sample.

### Immunoassays

All immunoassays were performed according to the manufacturers' protocols using plasma samples collected from the ECMO circuit study and quantified using the standards provided in the assay kits. The LegendPlex kits were read on a Cytoflex cytometer (Beckman Coulter, USA), while the Enzygnost assays were read on a SpectraMax i3 microplate reader (Molecular Devices). All standard and test samples were processed in duplicate.

### Statistical Analysis

Data were analyzed using GraphPad Prism version 9.4.1 and are expressed as mean ± standard deviation (SD). Multiple group comparisons were performed using ordinary one‐way ANOVA, followed by Tukey's post hoc test. P values were categorized in the plots as follows: >0.1234 (ns), <0.0332 (*), <0.0021 (**), <0.0002 (***), <0.0001 (****). Sample sizes (n) were determined based on previous experience and are stated in figure captions.

## Conflict of Interest

Duke University has submitted patent applications on the concept of EXACT inhibitors and Dr. Sullenger and Dr. Yu are listed as inventors on these patent applications.

## Author Contributions

Experiments were designed and conceived by H.Y., G.P., and B.A.S. Experiments were performed by: H.Y., G.P., M.Z, J. Clancy, S.G., and J. Caiazzi. Collection of blood reagents was supported by G.P., R.R., and S.F. Synthesis and purification of oligonucleotides was supported by A.Y. Data were analyzed by H.Y., G.P., J.W.F., R.R., R.S., and B.A.S. H.Y. and B.A.S. conceived the idea and wrote the manuscript. B.A.S. acquired the funding, provided resources, and supervision. All of the authors contributed to editing of the manuscript and support the conclusions.

## Supporting information



Supporting Information

## Data Availability

The data that support the findings of this study are available from the corresponding author upon reasonable request.
