# Supplementary material for: An Aptamer‐Based EXACT Anticoagulant as a Sustainable, Animal‐Free Alternative to Unfractionated Heparin
Source: Adv Sci (Weinh). 2025 Oct 6;13(4):e09867. doi: 10.1002/advs.202509867 (PMC12822440; doi:10.1002/advs.202509867)
Supplement: Supplementary file 1 — Supporting Information [file ADVS-13-e09867-s001.docx]

Supporting Information for

**An Aptamer-Based EXACT Anticoagulant as a Sustainable, Animal-Free Alternative to Unfractionated Heparin**

Haixiang Yu,^*,[a,b]^ George Pitoc,^[a]^ Manqi Zhang,^[c]^ Jeffrey Clancy,^[a]^ James Frederiksen,^[a]^ Amy Yan,^[a]^ Samuel Francis,^[d]^ Rebecca Sullenger,^[e]^ Rachel Rempel,^[a]^ Susannah Gammell,^[a]^ Jillian Caiazzi,^[a]^ Bruce A. Sullenger^*,[a,f]^

^[a]^Prof. Dr. H. Yu, Dr. G. Pitoc, J. Clancy, Dr. J. Frederiksen, Dr. A. Yan, Dr. R. Rempel, S. Gammell, J. Caiazzi, Prof. Dr. B. A. Sullenger

Department of Surgery

Duke University

Durham, 27710 (USA)

E-mail: [bruce.sullenger@duke.edu](mailto:bruce.sullenger@duke.edu) (B. A. Sullenger)

^[b]^ Prof. Dr. H. Yu

Department of Biochemistry, School of Life Science and Technology

China Pharmaceutical University

Nanjing, 211198 (China)

E-mail: haixiangyu@cpu.edu.cn (H. Yu)

^[c]^ Dr. M. Zhang

Department of Medicine

Duke University

Durham, 27710 (USA)

^[d]^ Dr. S. Francis

Department of Emergency Medicine

Duke University Hospital

Durham, 27710 (USA)

^[e]^ R. Sullenger

School of Medicine

Duke University

Durham, 27710 (USA)

^[f]^ Prof. Dr. B. A. Sullenger

Departments of Pharmacology & Cancer Biology, Cell Biology and Biomedical Engineering

Duke University

Durham, 27710 (USA)

**Note S1.** A two-step steered molecular dynamics simulation of aptameric hirudins with different linker lengths was used to determine the shortest linker required to permit the simultaneous binding of HD1 and DAB motifs. In the first step, the aptamer motif of the EXACT inhibitor was restrained to its binding site, while the 3' end of the linker was pulled toward the active center of thrombin. We found that linker lengths of 8-16 nt could reach within 1 nm of the active center, which enabled the placement of the conjugated dabigatran in the active site (**Fig. S1a-c**). Further shortening of the linker increased the distance between the active site and linker terminus, which disfavored bivalent binding. In the second step, while maintaining the steer of the linker terminus, the restraint on the aptamer was released, allowing the entire inhibitor to equilibrate. We found that only when the linker length was equal to or greater than 12 nt, the root mean square deviation (RMSD) of the HD1 motif was less than 0.5 nm from the original structure (**Fig. S1b-c**). The RMSD increased with the shortening of the linker, correlating with the distortion of the HD1 motif structure (**Fig. S1d**). When the linker length was reduced to 3-nt, the aptamer motif was completely pulled away from thrombin's exosite I. Based on these results, a poly (12) A linker was used to construct the aptameric hirudin.

**Table S1.** **All oligonucleotide sequences used in this work.**

| Sequence ID | Sequence (5' → 3') |
| --- | --- |
| HD1 | GGTTGGTGTGGTTGG |
| HD1-12A-DAB | GGTTGGTGTGGTTGGAAAAAAAAAAAA-DAB |
| HD1-12mA-DAB | GGTTGGTGTGGTTGG^m^A^m^A^m^A^m^A^m^A^m^A^m^A^m^A^m^A^m^A^m^A^m^A-DAB |
| HD1-12dmA-DAB | GGTTGGTGTGGTTGGA^m^AA^m^AA^m^AA^m^AA^m^AA^m^A-DAB |
| HD1-12dmA-NH_2_ | GGTTGGTGTGGTTGGA^m^AA^m^AA^m^AA^m^AA^m^AA^m^A-NH_2_ |
| AO | TTTTTTTTTTTTCCAACCACACCAACC |
| HD22-7A-DAB | DAB-AAAAAAAAGTCCGTGGTAGGGCAGGTTGGGGTGACT |

**
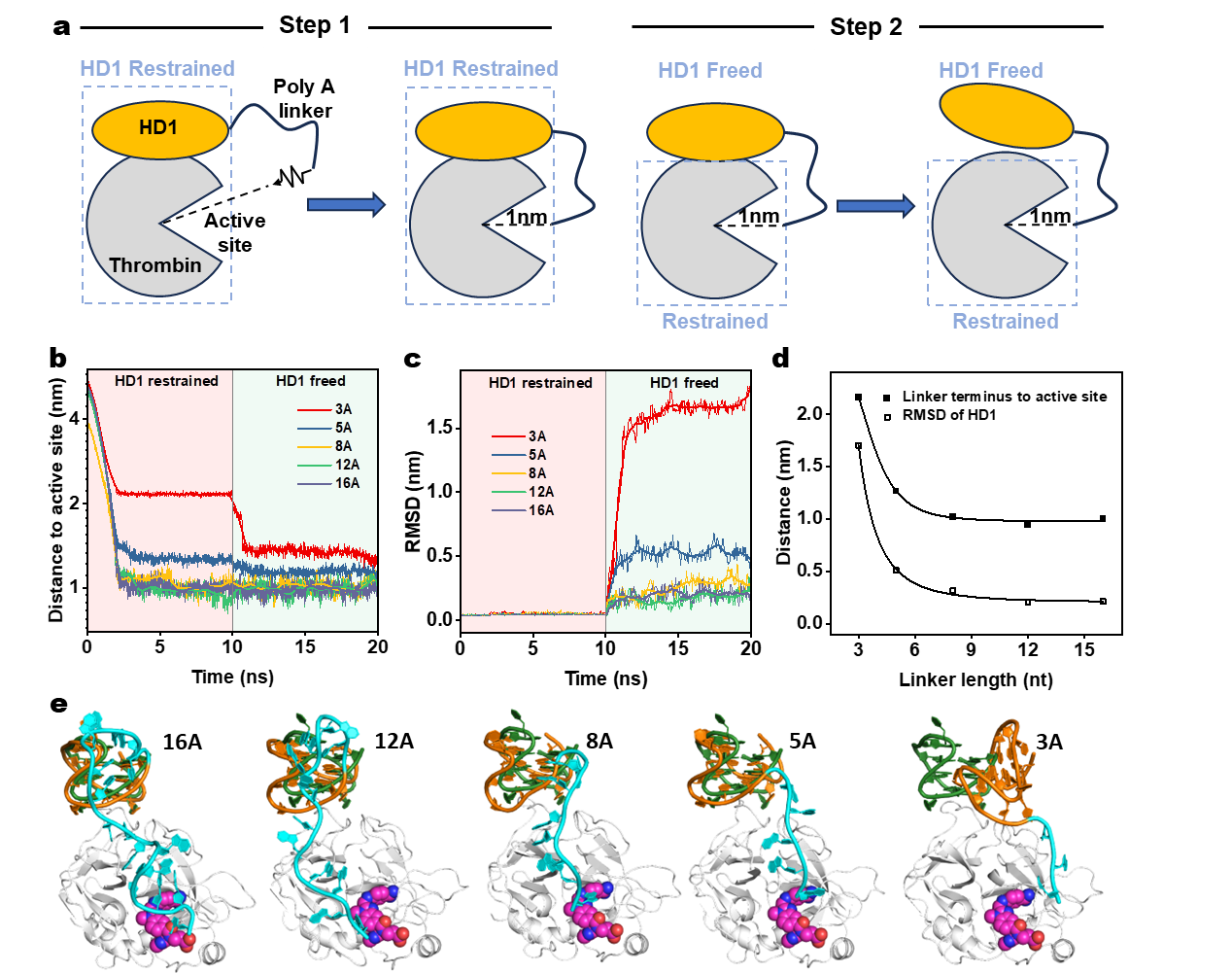
**

**Fig. S1. Optimizing the linker length of aptameric hirudin using molecular modeling. a. Schematic of the two-step molecular dynamics simulation. b.** Distance between the linker terminus and the thrombin active site during the simulation. **c.** RMSD of HD1 domains from its original structure during simulation. **d.** Summary of linker terminus-active site distances at the end of the first step and RMSD of the HD1 domains at the end of the second step. e**.** final complex structures after simulation, showing thrombin heavy chain (white), HD1 (orange), linker domain (cyan), and dabigatran (magenta). The reported HD1-thrombin structure (PDBID 4dii) is shown in green.

**Fig. S2.** Inhibition of free and clot-bound thrombin by dabigatran (2 µM), HD1-12A-DAB (2 µM), and ATIII (2 µM) + UFH (10U/mL) was tested in the fluorogenic peptidyl substrate cleavage assay. Error bars indicate the standard deviation of three independent measurements.

**
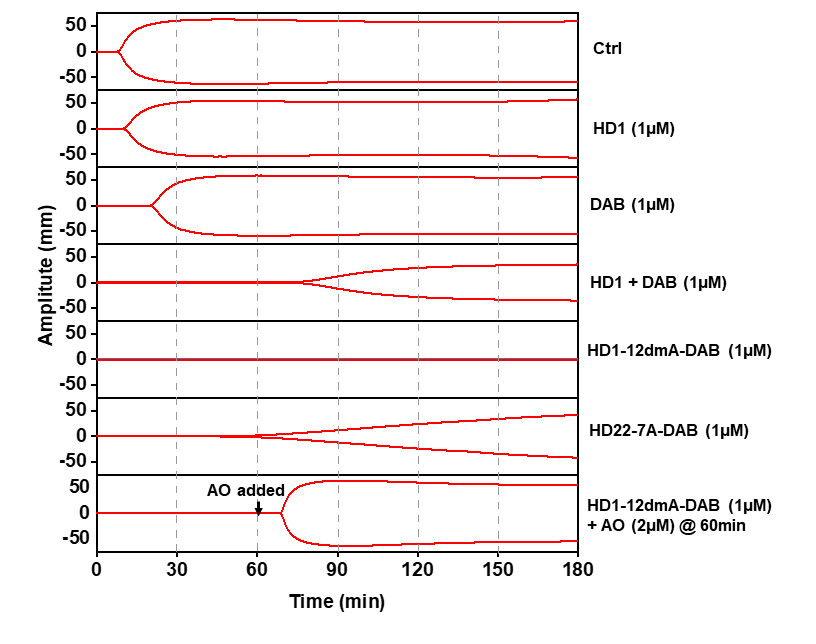
**

**Fig. S3. Representative** TEG clot tracing of human whole blood treated with HD1-12dmA-DAB compared with other anticoagulants. A higher amplitude (curve opening) indicates a higher clot stiffness. HD1-12dmA-DAB showed the highest anticoagulant activity, and no amplitude change was observed over 180 min. In the antidote study, AO was added 60 min after the test, and rapid blood clotting was observed.

**
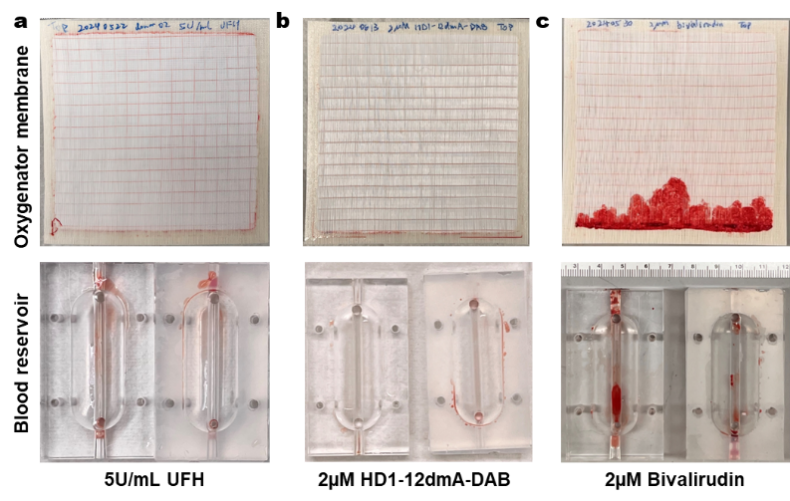
**

**Fig. S4. Representative images of oxygenator membranes (top) and blood reservoirs (bottom) after ECMO circuit experiments with 5 U/mL UFH (a), 2 µM HD1-12dmA-DAB (b), and 2 µM bivalirudin (c). Significant clots were observed only in the bivalirudin-treated group.**

**
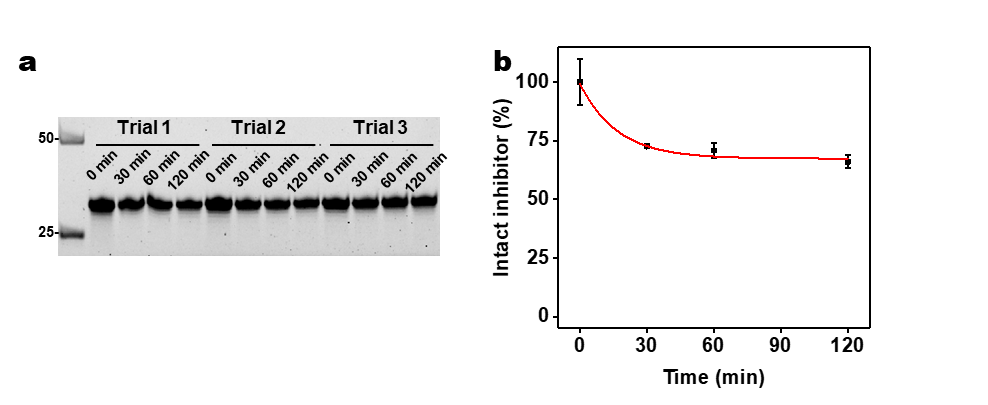
**

**Fig. S5. Stability of HD1-12dmA-DAB in three independent ECMO circuit experiments. a.**
HD1-12dmA-DAB was extracted from plasma samples collected before (0 min) and at various time points (30, 60, and 120 min) from *ex vivo* circuit experiments and analyzed via PAGE. No degradation products were observed. **b.** The concentrations of HD1-12dmA-DAB in samples were determined by band intensity. The average concentration of HD1-12dmA-DAB in the 0 min samples was normalized to 100%. Error bars indicate the standard deviation of three trials.
